# Barriers and facilitators to refugees, asylum seekers and people experiencing homelessness accessing non hospital based care: A mixed methods systematic review protocol

**DOI:** 10.12688/hrbopenres.13671.1

**Published:** 2023-02-23

**Authors:** Laura Fitzharris, Emer McGowan, Julie Broderick

**Affiliations:** 1 School of Medicine, Trinity Centre for Health Sciences, St. James Hospital, Dublin, D08W9RT, Ireland

**Keywords:** Marginalised groups, people experiencing homelessness, refugees, asylum seekers, access to non-hospital based care, primary care, community care, barriers, facilitators, social determinants of health, social exclusion, migrant groups, health equity, mixed methods systematic review.

## Abstract

**Context**: Social exclusion is characterised by and represents a form of disadvantage and marginalisation of vulnerable groups of people in society, who cannot fully participate in the normal activities of daily living. Socially excluded groups consist of, but are not limited to the following groups: people experiencing homelessness, asylum seekers and refugees. People from socially excluded groups have complex healthcare needs including infectious and non-communicable diseases. People from socially excluded groups tend to present more to the acute hospital setting as emergency presentations. Little is known about barriers and facilitators experienced by these groups to accessing non hospital based care.

**Objectives**: This mixed methods systematic review, will critically examine the concept of barriers and facilitators for refugees, asylum seekers and people experiencing homelessness, to accessing non hospital based care.

**Methods:** This methodological review will follow the Joanna Briggs Institute guidance for conducting mixed methods reviews. The following databases will be searched: Central Medline, PubMed, Embase, CINAHL, and the Cochrane Library. Relevant grey literature will be included. Title and abstract screening, followed by full-text screening will be undertaken independently by two reviewers. The Joanna Briggs Institute extraction tool will be adapted for data extraction.

**Discussion:** This mixed method review will comprehensively evaluate quantitative and qualitative data, synthesise both barriers and facilitators and follow a systematic approach through establishing use of mixed methods research across a number of marginalised groups, and how they affect accessing non hospital based care. It will explore conceptual models of access to healthcare and how they influence these factors.

## Introduction

Social exclusion is defined by the Council for the European Union as a “dynamic multi-dimensional process, whereby it involves the lack or denial of resources, rights, goods and services, and the inability to participate in the normal relationships and activities, available to the majority of people in society” ^1^. It affects both quality of life of individuals and the equity and cohesion of society as a whole ^2^. Social exclusion can distance individuals from educational opportunities as well as social and community networks and impact access to healthcare ^3^. Socially excluded groups consist of, but are not limited to the following groups: people experiencing homelessness (PEH), asylum seekers and refugees ^4^. Existing literature suggests the concept of social exclusion recognises the complex medical, psychosocial needs and health inequalities experienced by those in marginalised populations ^5^. People from socially excluded groups often have complex healthcare needs yet frequently report negative experiences accessing mainstream health services. The Inverse Care Law, that is, “the availability of healthcare is inverse to the health needs of the population”, (Hart, 1971) ^6^ is often applicable to PEH, asylum seekers and refugees, as these individuals encounter barriers when accessing healthcare services. 

In Europe the number of PEH has increased by 70% over the past 10 years and increasingly comprises of migrants, younger people, women, and families ^7^. In the UK PEH face up to 12 times higher mortality rates compared to the general population ^8^. In the USA, the average age life expectancy of the homeless population is 50 years old ^9^. PEH are two-to-five times more likely to die prematurely, with some studies reporting the highest risk among young people and women ^10^, with mortality related to a complex interaction of multiple co-morbidities, communicable diseases and substance abuse ^11^. PEH tend to have complex health needs across a wide range of health conditions namely; infectious diseases, non communicable diseases such as cardiovascular disease and diabetes as well as higher fraility scores with accelerated ageing compared to the general population ^12, 13^. These groups of individuals also report higher rates of self-harm, anxiety and depression ^14^. Psychiatric disorders play a significant role in determining engagement with health services and the relationship they play in compounding homelesness ^15^. 

Refugees and asylum seekers are another socially excluded group who have poorer health than the general population. Research indicates these groups are more susceptible to diabetes, communicable diseases, infectious diseases such as HIV and TB, health issues concerning women and children, work related health hazards, and poor mental health ^16, 17^. Many refugees have poor self-perceived general health ^18, 19^ and psychological well-being ^20, 21^. This is due to a multitude of factors namely; patterns of disease in their countries of origin (which can include a higher prevalence of communicable diseases), poor living conditions from the country to which they fled from, and the psychological stresses of travel and migration ^22^. Evidence suggests that refugees present with mental health and trauma symptoms, notably depression and post-traumatic stress disorder (PTSD) ^23^. Refugees also experience a high burden of malnutrition, anaemia, and infectious diseases ^24^. In the post migratory phase of refugees arriving to their host country, risk factors and protective factors relating to stressors of arrival and settlement in the host country have been identified ^25.^ These include individual-level factors relating to indicators of vulnerability (such as being of female gender or having a single parent, as well as family and societal factors (such as level of financial and social support). Furthermore, children and adolescents are impacted by mental health problems of the main caregivers, who may experience stresses related to trauma, migration, and resettlement ^26, 27^. Recognising these vulnerabilities and how they vary amongst marginalised groups over time (from the perspective of the social determinants of health), affects access to healthcare. 

Social determinants of health (SDOH) are non-medical and social factors that impact health outcomes ^28^. Social exclusion due to its multidimensional nature, is mentioned as one of the (SDOH) and one of the major driving forces of health inequalities in the world ^29^. SDOH can be grouped into five domains: economic stability, education, social and community context, health and health care, and built environment ^30^. They are directly related to poor health status; whereby persons who live in communities with limited access to health facilities, educational opportunities and poor quality housing, experience greater health disparities than those of higher socioeconomic status ^31^. The SDOH also include “upstream” factors, such as social disadvantage, risk exposure, and social inequities, playing a fundamental causal role in poor health outcomes, and health disparities ^32^. Most evidential is that a social gradient exists in health whereby health is progressively better the higher the socioeconomic position of communities ^33^ and people who are socially excluded experience multi-layered barriers and facilitators to accessing healthcare ^34^.

PEH experience higher readmission rates to hospital ^35^. PEH typically attend the emergency department more often than non-homeless persons. Older PEH (>50), often lack social support and do not explicitly express their own healthcare needs, resulting in higher cases of acute care ^36^. PEH are influenced significantly by SDOH, such as housing insecurity, unemployment, and limited social support ^37^. For example PEH are known to be 40 times less likely to be registered with a mainstream general practice compared with the general population ^38^. It is known that this population disproportionately attend the accident and emergency department, with a visit rate 60 times that of the general population ^39^. There is relatively lower uptake of primary care and preventative healthcare services in this group, which may suggest that health is often not a priority until a crisis point is reached, but overall little is known about the precise reasons why PEH don’t tend to present as well for care outside emergency settings.

Refugees and asylum seekers have also been found to demonstrate different patterns of health services access and utilisation. The health of refugees on the move in Europe is jeopardised by poor living circumstances and access to healthcare, due to language difficulties, communication, and attitudinal discrimination ^40^. It has been noted that most host countries had either a higher or lower service utilisation pattern amongst these groups, depending on the type of health service required, for example in the UK, increased use of emergency services similar to PEH and underutilisation of specialist services and primary care ^41^. Morris *et al*. (2009) ^42^, listed practical barriers impeding access to healthcare including inadequate information on availability of services, restricted access to transport and culturally insensitive care. The legal status for those arriving to their host country is a significant barrier for many of these individuals in accessing health services in Europe, and that where entitlements do exist, that there are pathways in place for ensuring that they are respected in practice ^43^. Asylum seekers and refugees often have more limited comprehensive health literacy ^44, 45^. In addition, many refugees refrain or are more reluctant to seeking primary healthcare such as participating in health promoting and disease preventing activities ^46, 47^. Suggested reasons for this include factors such as difficulty navigating and adapting to new healthcare services, systems, and language barriers, all playing significant factors to accessing healthcare in these groups ^48^. So too is comprehensive health literacy. Limited comprehensive health literacy has been found to be influenced by SDOH such as older age (multiple co-morbidities) and lower levels of education (including capacity for informed decision making) ^49^. In addition, comprehensive health literacy has been found to be associated with various health outcomes in chronic diseases and the benefits from disease preventing efforts ^50^. In a study mapping the health needs of refugees and migrants in 10 European countries, Lebano *et al*. (2020), highlighted the importance of facilitating effective healthcare provision including non-hospital based care particularly with the presentation of chronic diseases and mental health conditions ^51^. A recently published paper in the Lancet also highlighted this need ^52^. In it, the authors reported on the European Centre for Disease Control’s guidance in the area of universal health care to allow easier healthcare access for these groups. They outlined a roadmap and design to remove barriers to access, emphasising accessibility that includes primary care and universal health care.

Health care systems as a whole, can be considered a SDOH, in particular how they are used by vulnerable and marginalised groups and can in fact prolong and continue the extent of inequities in accessing healthcare ^53^. This work has been publicised and disseminated by other large organisations such as the World Health Organisation and prominent authors on this topic, such as Michael Marmot writing in 2008, that he himself regarded health-care systems, as a social determinant of health ^54^. The structure and organisation of health systems, including their operationalisation as shaped by government and social policy, can have a deep and weighted influence on the ability of particular groups to access health care. This is particularly of importance for individuals with low health literacy, different cultural backgrounds, or language barriers. These aforementioned factors can lessen their capacity and ability to access healthcare ^55^. This indicates that healthcare systems can in fact, magnify or alleviate the impact of inequities caused by the SDOH ^56^. This is why research on identifying various health care systems, how they operate, and the scope of primary care to meet the health needs of marginalised groups has become increasingly important ^57^. 

Linking conceptual models (a *model that is constructed to demonstrate or explain a concept or situation*), with SDOH, is important to understand and create a larger picture of the many layers to accessing healthcare ^58^. Conceptual models for accessing healthcare recognises access as the outcome of a process involving the interplay between the characteristics of the health care system (accessibility, affordability) and of potential users’ health literacy and beliefs, often times weakened and shaped by health care related public policy and planning efforts ^59^. Several dimensions to accessing healthcare exist; including availability, acceptability, affordability, transportation, as well as health literacy, capacity and the communication skills needed to engage with healthcare providers to facilitate decision making ^60^. Although the ability to access healthcare, depends on the structural circumstances, so too is the navigation process; a set of dynamic processes through which individuals can access, seek and obtain care based on their healthcare needs ^61^. These processes are influenced by certain conditions namely: biomedical, psychosocial, economic, and environmental. These conditions are influenced by factors such as race, ethnicity, cultural norms, expectations, and opportunities such as the availability of specialist services. Identifying conceptual models provides a better description and illuminates the greater picture of the ‘access concept’, especially the crucial linkages among the various dimensions to accessing healthcare, and factors involved. It presents a comprehensive conceptual framework for evaluation. The ‘behavioural ecological framework’ is one such concept. In a paper by Sallis and Owen 2008, the authors discussed this relatively new conceptual framework for examining healthcare access and navigation from a behavioural-ecological perspective ^62^. The core concept of this ecological model is that behaviour has multiple levels of influences, often including ‘‘intrapersonal (biological, psychological), interpersonal (social, cultural), organisational, community, physical and environmental”. This highlights the notion that an individual’s social environment and environment in which they reside are influential factors in healthcare access and navigation. It also describes in detail how various factors such as education, environment, and the individual’s medical needs are at play in these vulnerable groups accessing care, with particular emphasis on vulnerable populations who are disproportionately affected by health disparities.

The importance of including socially excluded groups in health and social care research has gained momentum and recognition over the past number of years ^63^ and is underpinned by recent UK government policy and the most recent WHO report in 2022 ^64^. This report highlighted the need to improve access and quality of services for socially excluded groups with particular emphasis on effectively integrating socially excluded groups into primary health care. The authors emphasised the need for a shift towards health promotion and disease prevention by addressing SDOH; thus improving access. 

These vulnerable groups with chronic diseases, exposed to layers of barriers to healthcare access, means participation in accessing healthcare is more difficult ^65^. Social exclusion is a complex and multidimensional concept that affects how these vulnerable groups access healthcare. Previous research has aimed to measure social exclusion at an individual and population level. One such research paper was that of O Donnell *et al*. (2002). In this paper the authors engaged with various stakeholders from academic, clinical and political backgrounds, to learn how they defined and conceptualised social exclusion ^66^. The authors concluded that the overall definition of social exclusion was the “experience of lack of opportunity, or the inability to make use of available opportunities, thereby preventing full participation in society”. From this research a new model was developed consisting of three main areas to illustrate the concept of social exclusion. These three areas were opportunities (to participate in society), social outcomes and influencing factors (mixture of individual characteristics and complex health needs, including the intergenerational effects of disadvantage). 

Given the apparent under-utilisation of non-hospital based services in socially excluded groups, the increased use of accident and emergency settings in PEH, different patterns of health services access and utilisation amongst asylum seekers and refuges, there is a need to further explore barriers and facilitators to accessing non hospital based services alongside underlying conceptual models (related to access to healthcare) among these groups. Most previous studies to date have concentrated on qualitative data only, however it is the view of the authors, that this MMSR will critically explore these factors to better understand comprehensively (through synthesising qualitative and quantitative data, mixed data), their effects on marginalised groups accessing non hospital based care while utilising the conceptual models of access to healthcare. 

## Protocol

### Review objectives

The purpose of this mixed methods systematic review is to:

Comprehensively synthesize the best available evidence based on quantitative and qualitative data to develop concepts underpinning facilitators and barriers to refugees, asylum seekers and persons experiencing homelessness.

The effects of these factors on accessing non hospital based care amongst these marginalised groups. 

Provide a comprehensive narrative framework on stakeholder’s experiences and perceptions of these marginalised groups accessing healthcare.

### Research question

To what extent are the concepts of barriers and facilitators are defined and how do these concepts underpin theories in the context of accessing non hospital based care to people experiencing homelessness, refugee and asylum seeker groups?

What are the facilitators and barriers to people experiencing homelessness (PEH), refugees and asylum seekers to accessing non hospital based care?

 What are the experiences and perspectives of stakeholders (GP’s, health care professionals, voluntary care agencies, charity organisations, caregivers, people experiencing homelessness, asylum seeker, refugees, those managing health systems), involved in accessing non hospital based care?

### Protocol methods

A convergent segregated mixed methods systematic review will be conducted. This protocol was designed under the guidance of the methodological framework provided by the Joanna Briggs Institute (JBI) reviewer’s manual and reporting guidelines on conducting a MMSR ^67^. It also adheres to the PRISMA for systematic review protocols (PRISMA-P) ^68, 69^.

## Types of studies to be included

A broad representation of primary evidence based data (qualitative, quantitative, mixed methods) will be included. Studies involving stakeholders such as policy makers, health care providers, GP, health care professionals, volunteers, care providers, health systems managers, clinicians, patient representatives, marginalised groups in society will be included.

### The PICO format for the quantitative review and the SPIDER format for the qualitative review will be used

***Quantitative (observational, prospective, cross sectional).***

Qualitative (Interviews, focus group studies, surveys, questionnaires) and mixed methodology studies, published in English in developed countries will be reviewed. Studies relating to non hospital based care (i.e. not relating to, associated with, or occurring within a hospital); including primary care, out reach services, stand alone clinics, day centres, open access services, voluntary agencies, etc.

This will include studies using both a qualitative and quantitative (cross sectional, prospective) description of barriers and facilitators to accessing health care services for these specific marginalised groups identified in this study. It will also include longitudinal qualitative studies exploring facilitators and barriers to accessing the healthcare system, explorative studies on perspectives of refugees, asylum seekers, and people experiencing homelessness in accessing non hospital based health care. Studies relating to non hospital based care (i.e. not relating to, associated with, or occurring within a hospital); including primary care, out reach services, stand alone clinics, day centres, open access services, voluntary agencies. 

### Inclusion criteria

Qualitative, quantitative and mixed methods studies which investigate access of specified marginalised groups (limited to PEH, refugees and asylum seekers) to non-hospital based care for a physical and/or mental health condition will be included. Non hospital based care means not relating to, associated with, or occurring within a hospital, so eligible studies will relate to primary care (PC), primary health care (PHC), and community care (CC) or other non-hospital based setting such as GP practices, pharmacies, dental surgeries, ophthalmic services, screening and immunisation services. The age group included will be adults > 18 years and adolescent groups aged 13–18 years. If studies contain mixed age-groups, for instance children <13 years or marginalised groups other than those included in this review-data must be available separately for the populations of interest, or if mixed, then >80% of the population must be the population of interest to this review. Studies have to be based in Organization for Economic Co-operation and Development (OECD) countries. Full text articles, grey literature including academic papers, research and committee reports, government reports and conference papers in English will be included. There will be no restriction regarding the year of publication of studies.

### Exclusion criteria

Exclusion criteria includes (a) abstracts, editorial letters, commentaries, conference proceedings, (b) method papers or protocols, (c) studies on children under age of 13, family studies where 80% of included participants were children, (d) non english language studies, (e) studies that have no health related function, (f) studies that report no evaluation of the barriers and facilitators in accessing health care such as case studies, (g) literature that does not contain original data or analysis of acute hospital based care for example those that present to accident and emergency services, (h) individuals who are not asylum seekers, refugees or PEH, (i) any services provided in hospital settings and tertiary settings, (j) studies of low methodological quality through performing sensitivity analyses, exerting undue influence. 

The Mixed methods systematic review (MMSR) will be guided by the application of inclusion and exclusion criteria during the screening of retrieved papers. The PICO format will also aid this as outlined below. 

### Population/participants

People who are homeless (by definition) – street, statutory, hidden or concealed. The European Federation of National Organisations working with the Homeless (FEANTSA) has developed a European Typology of Homelessness and housing exclusion (ETHOS) as a way to improve the understanding and measurement of homelessness in Europe, providing a common “language” for international discussion on homelessness. These include rooflessness, houselessness, living in insecure housing and living in inadequate housing ^70^.

Refugees – The UNHCR convention of 1951 defines refugees as persons who have a ‘‘well-founded fear of being persecuted for reasons of race, religion, nationality, membership of a particular social group or political opinion, is outside the country of his nationality and is unable or, owing to such fear, is unwilling to avail himself of the protection of that country; or who, not having a nationality and being outside the country of his former habitual residence as a result of such events, is unable or, owing to such fear, is unwilling to return to it” ^71^. 

Asylum seeker: A person who has left their own country to seek sanctuary in another country and has applied for asylum – the right to be recognized as a refugee and receive protection and material assistance. As defined by UNHCR – UN agency of Ireland, those “persons in the process of awaiting to be granted refugee status are referred to as ‘asylum seekers” ^72^. 

### Conditions/domains being studied

Primary care: Primary care is the provision of integrated, accessible health care services by physicians and their health care teams who are accountable for addressing a large majority of personal health care needs, developing a sustained partnership with patients, and practicing in the context of the community. The care is person-centred, team-based, community-aligned, and designed to achieve improved quality of care, at a lower cost. Together they form a framework where patients can access efficient, equitable, and effective primary care services of the highest quality leading to better care, better health, and lower costs. This definition is in accordance with the Alma Ata Declaration on Primary Care, WHO – UNICEF, 1978 ^73^. A modern day definition is ‘‘first contact, continuous, comprehensive and co-ordinated care provided to individuals and populations undifferentiated by age, gender, disease or organ system” ^74^.

Non hospital based care identified in this review will include primary care services such as; primary care centres, mobile health clinics, stand-alone clinics, open access GP and nurse led homeless services, day centres, and other voluntary agencies. This review covers topics on healthcare accessibility, homelessness and health experiences of those involved in these services. These include demographic characteristics, physical and mental health status. 

The refugee experience is typically divided into two phases: pre-migratory and post-migratory. Pre-migratory experiences may include witnessing or experiencing violence, torture, murder, physical and emotional trauma, or a combination of these to the individual or family. It also includes the long and arduous journey to the destination country. The post-migratory phase may present challenges such as linguistic and cultural barriers, poverty, discrimination, and adapting to a new and foreign environment ^75^. For this purposes of this study, the conditions and domains being studies are in the post migratory phase. 

### Exposures

These include those experiencing homelessness, refugees, those seeking asylum internationally and those in direct provision. Experiences and perceptions of stakeholders and marginalised groups to barriers and facilitators to accessing non hospital based care.

There is a theoretical understanding that the definition of access to healthcare is taken from ‘Levesque’s access to care framework’, whereby access is described as a ‘‘two-sided relationship with service provisions and service user abilities both affecting the process of access to healthcare” ^76^.

## SPIDER for the qualitative evidence synthesis

**Sample:**                              Clinicians, GP’s, health care professionals

**Phenomenon of Interest**:  Barriers and facilitators to accessing non hospital based care

**Design**:                               Focus groups, interviews, questionnaires, surveys

**Evaluation outcomes:**      Beliefs, attitudes, opinions, views, experiences, perceptions, feelings, understanding.

**Research type**:                 Qualitative studies, and mixed methods studies with primary qualitative data collection. 

## Search strategy

### Databases search and studies selected

***Two independent reviewers will conduct a search using the following electronic databases:***

Central Medline, PubMed, EMBASE, CINAHL, Scopus, Web of Science, BMJ Best Practice, AMED, OVID, Psychlit, Cochrane Library and EBSCO. Relevant studies will be searched to further identify articles that meet our inclusion criteria using Google scholar search engines.

Key words and controlled vocabulary will be used for suitable search terms and potential studies. See Box 1 for the full search strategy.

**Box 1. Search strategy**

'forced migrant'/ OR 'undocumented immigrant'/, (displaced or undocumented) (person* OR people OR immigrant* OR population* OR migrant*)):('forced migrant*' OR ‘migrant worker*’ OR 'forced migration*' OR refugee* OR 'asylum seeker*' OR 'unaccompanied minor*' OR 'illegal immigrant*' OR 'unauthorized immigrant*' OR 'undocumented alien*' OR 'undocumented immigrant*'):homelessness'/exp OR 'homeless person'/exp OR 'homeless youth'/exp (homeless* OR ‘Housing insecur*’), 'community care'/de OR 'community based rehabilitation'/exp OR 'community health nursing'/((Communit* OR community-based) (rehabilitation OR care OR healthcare OR 'health care' OR nurs*'home care'/exp ((Home OR home-based OR domiciliary OR domestic) (care OR healthcare OR 'health care', primary health care'/exp OR 'family medicine'/exp OR 'general practice'/exp OR 'general practitioner'/exp, (primary care OR healthcare OR 'health care'))((general OR family) (practice* OR practitioner* OR physician*)): (Barrier* OR facilitator* OR impediment* OR obstacle* OR deterrent* OR difficult* OR enabler* OR promot* OR benefit* OR burden* OR aware* OR equality OR inequality): Ability OR able) NEAR/2 (perceive OR seek OR reach OR engage)):Accomodat* OR Availability OR approachab* OR appropriate* OR acceptability OR affordab* OR appropriate* OR 'culturally responsive', 'health care utilization'/exp OR 'health care access'/exp, ((Access* OR accessibil* OR utilis* OR utiliz*) NEAR/5 (care OR healthcare OR 'health care' OR 'health service*' OR support* OR therap* OR rehab* OR service* OR physical*

Targeted searches on asylum seekers and refugees and persons experiencing homelessness will be conducted with papers identified informing subsequent targeted searches. Checks of reference lists of retrieved articles and citation searches will be conducted. 

Databases will be searched from inception to present. The search strategy is planned by the primary author and supported by a librarian with systematic review experience using the planned structured search outlined above. Two independent reviewers will search the databases using the search items and keywords outlined. Reference lists of related articles and relevant reviews including grey literature will be checked.

### Study records

Research articles identified from the literature search will be uploaded to Endnote X8, a citation manager system. Duplicates will be removed using the “remove duplicates” function, and manual screening of the results will be conducted to ensure accuracy. Titles and abstracts of the identified articles will then be exported to Covidence, an electronic web based software designed to support article screening and allows collaboration between reviewers during the study selection process.

### Data collection and extraction

Two reviewers will independently extract data from papers included in the review using standardised data extraction tools adapted from JBI. Specific data about the population, setting, context, methods and outcome related to the review questions and specific objectives will be included. This will also include general information; (title, author(s), year, country of study, study characteristics (study design, aim, population, duration of study), risk of bias, quality assessment, outcomes, barriers, facilitators, (related to health outcomes), limitations, adverse effects (if any). We will pilot the data extraction form on a small number of studies to develop the final data extraction form. The reviewers will discuss key emerging themes related to the objectives of the review and agreed theme level for data extraction. The data extracted will include specific details on marginalised groups accessing non hospital based care, phenomena of interest, populations described, study methods and outcomes of significance to the review question. Relevant verbatim data from research participants included in studies will be extracted to illustrate findings. 

### Methodological quality of studies

Studies that have passed full-text screening will be assessed with the appropriate JBI quantitative and qualitative checklists (depending on the specific study type) for critical appraisal. The qualitative and quantitative parts of mixed-methods studies will be appraised separately.

The reviewer will comment on the quality of evidence in the review and take it into consideration during analysis and discussion. All search results for each study will be independently reviewed for eligibility by two reviewers. It will be done in two phases. In the first phase, two reviewers will screen the articles identified by the search title, abstract for eligibility. The two reviewers will compare the lists of identified articles as to whether they met the inclusion criteria. The second reviewer will randomly select 10% of the abstracts against the inclusion criteria to investigate agreement. Articles thought to partially meet the inclusion criteria will be discussed to make final decision. Disagreements during initial screening will be resolved through discussion or will be resolved through arbitration from a third reviewer. 

### Assessing quality of evidence

The quality criteria for this review will be assessed using the JBI critical appraisal tool for systematic reviews. This allows for a more ‘integrative’ review of all of the research studies included in this systematic review. The authors chose this method of critical appraisal for systematic reviews using the JBI methods of review as it focuses on basing the best available evidence, and is adaptable to the complexity of the research question to generate evidence appropriate to the issue. Such a systematic review seeks to synthesize and summarize existing knowledge. This allows appropriate decision-making that considers the feasibility, appropriateness, meaningfulness, and effectiveness of the chosen studies. Using the best available research in the context of this systematic review on access to healthcare amongst these specified groups and the expertise and professional judgment of the independent reviewers in this review, these two factors will play a role in ensuring quality in this process. It will also identify the quality of studies as low medium or high quality. This will also help and reconcile any difficulties in decisions to include or exclude studies. Results of the quality assessment for each study will be documented and taken into consideration during the analysis and discussion of the review.

For example the qualitative component of the analysis phase will involve the identification and classification of the facilitators and barriers to accessing healthcare as described above. The studies will be grouped into in order to classify the main facilitators and barriers emerging from this field of data. Thus a narrative approach to data synthesis will be chosen. Study criteria to assess quality of studies will include: Title, abstract, context of problem statement, purpose of research question, study design, participation selection, sampling size, description of sample, research teams’ reflexivity, method of data collection, setting of data collection, audio, visual, interview recording, description of coding, study findings and data analysis. 

### Narrative synthesis and analysis of data

Using updated JBI methodological guidance for conducting a mixed methods systematic review, the reviewers will take a convergent approach to synthesis and integration whereby the specific method utilized is dependent on the nature/type of questions that are posed in this systematic review ^77^. As the research questions focuses on different aspects or dimensions of a particular phenomenon of interest in marginalised groups, i.e. both quantitative (age, context, accommodation, physical health status, language) and qualitative aspects (facilitators, barriers, attitudes and perspectives), the convergent approach will be taken, which involves independent synthesis of quantitative and qualitative data leading to the generation of quantitative and qualitative evidence, which are then integrated together. Covidence will be used for title and abstract screening as well as full text screening as the primary screening and data extraction tool for conducting mixed methods reviews. This is important to obtain an overarching picture of the inherent complexities of this research question associated with marginalised groups. This approach also aligns with the typology as developed by Hong *et al*. (2018) due to the nature of the research question posed ^78^. 

A convergent qualitative synthesis approach will be used to transform quantitative and mixed methods studies into qualitative findings and subsequently pooled with the qualitative narrative synthesis in tabular form using the Bayesian approach described by Crandell *et al*. (2011) ^79^. In this process, outcomes deemed conceptually similar will be mapped to a framework in theme and then data will be coded for each concept. 

Quantitative data will be extracted and interpreted into a narrative description thus allowing integration with qualitative data. The individual strands of the mixed methods studies will be independently analysed. Resulting qualitative data will be pooled for thematic analysis conducted by the primary reviewer. This process involves qualitising quantitative outcomes of data by generating codes, searching for themes, reviewing themes, defining and naming themes and finally reporting findings in a narrative synthesis. The purpose of this discussion is to contextualise the review findings with refugees, asylum seekers and PEH to examine facilitators and barriers to accessing non hospital based care. 

### Subgroup analysis

Groups identified include PEH, asylum seekers and refugees. There may be a specific subgroup analysis involved for example migrants who experience homelessness themselves. 

## Discussion

Access to healthcare services is recognized as an important social and public health challenge. In European countries and across the United States, there is an abundance of growing evidence of an increase in the awareness of social determinants, the impact they have and their relationship to health, in medical research over the past 10 years ^80^. 

Given the increased use of emergency settings in PEH, under-utilisation of primary care services, including variable patterns of utilisation of primary and specialist health care of refugees and asylum seekers, there is an underlying need to further explore the barriers and facilitators to accessing non hospital care in these marginalised groups. 

This mixed methods review (MMR) will comprehensively review quantitative data and qualitative data (experiences of stakeholders and health care providers) and specifically refugees, asylum seekers and PEH and synthesise both barriers and facilitators outlined and embrace a systematic approach through identification of mixed methods research. A mixed methods approach was chosen as using both qualitative and qualitative data will provide a more comprehensive insight into the best available data and evidence of the many complicated personal and structural barriers to healthcare access across a number of marginalised groups. It is also hoped to acquire a clearer understanding of the various barriers and facilitators to accessing healthcare in order to provide more comprehensive information for policy makers. The authors believe more explicit use of conceptual models in how these groups access healthcare as part of this MMSR, will help to maximise the usefulness of how barriers and facilitators operate in relation to accessing non hospital based care in these groups. 

## Study status

This study has been registered with Open Science Framework, subject to a peer review process, with a view to subsequent publication of a fully detailed mixed methodology systematic review by end of year 2023. Full text and abstract screening is currently underway. 

## Dissemination

It is hoped this protocol will be peer reviewed and approved with subsequent production of a fully detailed mixed methodology systematic review by end of year 2023.

## Data Availability

No data are associated with this article. Open Science Framework: ‘Barriers and facilitators to refugees, asylum seekers and people experiencing homelessness accessing non hospital based care’: A mixed methods systematic review protocol. https://doi.org/10.17605/OSF.IO/WRQ64 ^69^. Data are available under the terms of the Creative Commons Zero "No rights reserved" data waiver (CC0 1.0 Public domain dedication).
