# Supplementary material for: Barriers and facilitators to refugees and asylum seekers accessing non hospital based care: A mixed methods systematic review protocol
Source: HRB Open Res. 2023 Dec 19;6:15. Originally published 2023 Feb 23. [Version 2] doi: 10.12688/hrbopenres.13671.2 (PMC11007367; doi:10.12688/hrbopenres.13671.2)
Supplement: Peer review history

# Reviewer report: Reviewer response for version 2

**DOI:** 10.21956/hrbopenres.15137.r39402

**Published:** 2024-05-10

**Authors:** Anna Gordon

**Affiliations:** 1 University of Bristol, Bristol, UK

A thorough protocol to investigate an important and relevant topic. Well done! I look forward to reading the review. 

 Introduction is very thorough and clear. 

 A few small comments-

 1. In ‘Types of studies to be included’ paragraph 2 you mention studies ‘published in English in developed countries’. I noted following comments from a previous reviewer you are including ‘studies based in Member States of the European Council’ (as per your Inclusion  criteria). I agree with the rationale to use Member States of European Council based studies- perhaps update the previous paragraph to reflect this. 

 2. In the inclusion criteria, and  discussion you mention investigating access of refugees and asylum seekers. In ‘Population/participants’ you define these clearly. In the search strategy, however, you also seem to be including undocumented/illegal/unauthorised migrants too though. While I think this group is very important to include, I think you could specify them as their own sub-group as their legal status and therefore access to healthcare is so different than those seeking asylum, or with refugee status. You could also look at doing a subgroup analysis on this. I anticipate that there will be really minimal data on this population however, so it may not be possible.

Is the study design appropriate for the research question?

Yes

Is the rationale for, and objectives of, the study clearly described?

Yes

Are sufficient details of the methods provided to allow replication by others?

Yes

Are the datasets clearly presented in a useable and accessible format?

Yes

Reviewer Expertise:

Inclusion health with a focus on people experiencing homelessness, refugees and asylum seekers.

I confirm that I have read this submission and believe that I have an appropriate level of expertise to confirm that it is of an acceptable scientific standard.

# Reviewer report: Reviewer response for version 2

**DOI:** 10.21956/hrbopenres.15137.r37513

**Published:** 2024-04-10

**Authors:** Rebecca Farrington

**Affiliations:** 1 The University of Manchester, Manchester, England, UK

Thank you for considering all aspects of the review. I agree that the changes you have made do make the paper stronger and am happy to approve without further comment

Is the study design appropriate for the research question?

Partly

Is the rationale for, and objectives of, the study clearly described?

Yes

Are sufficient details of the methods provided to allow replication by others?

Yes

Are the datasets clearly presented in a useable and accessible format?

Not applicable

Reviewer Expertise:

I work with people seeking asylum and new refugees in primary care and mental health settings

I confirm that I have read this submission and believe that I have an appropriate level of expertise to confirm that it is of an acceptable scientific standard.

# Reviewer report: Reviewer response for version 1

**DOI:** 10.21956/hrbopenres.14950.r35436

**Published:** 2023-10-16

**Authors:** Patrick O'Donnel

**Affiliations:** 1 University of Limerick, Castletroy, Limerick, Ireland

The protocol sets out a comprehensive plan for approaching this research question of importance. Well done.

 I have some suggestions to consider:

- Good introduction section.
- The terminology of 'non hospital based care' may be confusing & make searching challenging. Primary care, GP care etc. are pretty straightforward and understood easily, but there are others. For example, would you consider community psychiatric clinics as part of this, even though they are considered secondary care services? Or newer services such as cardiology or care of the elderly community clinics, would they be included? Whatever you settle on, just be really clear in your inclusion criteria as this will help to streamline your search strategy.
- I feel you need some more explanation on the rationale for examining the issues of homelessness and migration together. You do a good job of explaining the reasons for looking at each of these groups individually, and the likely barriers and facilitators to access for each of them. It is clear that both of these groups have significant problems with access. It seems that you think for the most part though that the likely barriers and facilitators will not overlap ("Refugees and asylum seekers have also been found to demonstrate different patterns of health service access and utilisation.") - So where is the synergy in examining them together? Are you looking for common levers or barriers in order to suggest practice or policy change, or are you looking to contrast these?
- Good methodology / protocol section.
- What is the reason for excluding children <13 years?
- What is the rationale for using the OECD member countries? Are there expected similarities across health systems in the OECD? Why OECD versus the EU versus the WHO Europe Region? This is important because some of the barriers and facilitators discovered may relate to the structure and universality of primary care in the countries under scrutiny, as well as other factors. 
- You mentioned using ETHOS Typology as a guide for your inclusion criterion of  'homelessness', but as well as groups we traditionally consider as homeless, ETHOS includes people in accommodation for migrants, domestic violence shelters, penal institutions, couch surfing, halting sites, overcrowded settings etc., so you may need to narrow the criteria here.
- Comprehensive explanation of the extraction, screening and planned synthesis.

Is the study design appropriate for the research question?

Yes

Is the rationale for, and objectives of, the study clearly described?

Yes

Are sufficient details of the methods provided to allow replication by others?

Yes

Are the datasets clearly presented in a useable and accessible format?

Not applicable

Reviewer Expertise:

social exclusion, inclusion health, homlessness

I confirm that I have read this submission and believe that I have an appropriate level of expertise to confirm that it is of an acceptable scientific standard.

## References

[1] : Factors associated with multiple barriers to access to primary care: an international analysis. *Int J Equity Health* .2018;17(1) : 10.1186/s12939-018-0740-1 28 10.1186/s12939-018-0740-1 29458379 PMC5819269

# Response

**Published:** 2023-11-27

**Authors:** Laura Fitzharris

**Affiliations:** Trinity University, Ireland

Dear Dr. O’ Donnell,

 Many thanks for your feedback, it is greatly appreciated.  

**1. The terminology of 'non hospital based care' may be confusing & make searching challenging. Primary care, GP care etc. are pretty straightforward and understood easily, but there are others. For example, would you consider community psychiatric clinics as part of this, even though they are considered secondary care services? Or newer services such as cardiology or care of the elderly community clinics, would they be included? Whatever you settle on, just be really clear in your inclusion criteria as this will help to streamline your search strategy.**

- Thank you this is a very useful point, and we acknowledge that non-hospital-based care encompasses a broad array of services.  It is likely that the exclusion of acute hospital-based services will guide the inclusion of the appropriate services to this review. We will add the other mentioned community services such as elderly clinics and psychiatric clinics as part of non-hospital-based care into the protocol paper. The authors appreciate mental health is part of the many physical and psychological illnesses that ASR experience in their host and native countries and has an impact on access to healthcare. 

**2. I feel you need some more explanation on the rationale for examining the issues of homelessness and migration together. You do a good job of explaining the reasons for looking at each of these groups individually, and the likely barriers and facilitators to access for each of them. It is clear that both of these groups have significant problems with access. It seems that you think for the most part though that the likely barriers and facilitators will not overlap ("Refugees and asylum seekers have also been found to demonstrate different patterns of health service access and utilisation.") - So where is the synergy in examining them together? Are you looking for common levers or barriers in order** to suggest practice or policy change, or are you looking to contrast these?

  

- This is a most important aspect of the feedback and directly concurs with feedback provided by the other reviewer. We decided that we will only include the literature relevant to ASR.  We recognise PEH as a group, are another distinct entity with unique characteristics that deserve separate consideration with regards to healthcare utilisation patterns and considering both groups together is beyond the scope of this review paper.  

**3. What is the reason for excluding children <13 years?**

- We gave this age cut off a lot of consideration and chose it, as children > 13 years of age who are asylum seekers or refugees have caring responsibilities and are therefore considered with adults in other literature including ASR.  For instance the cut off chosen is the same as for two large scale systematic reviews by Fazel et al in (2005) and (2012),  published in the Lancet which explored the effect of Pre-migratory adverse events and the complexities of post-migration settlement. 

**4. What is the rationale for using the OECD member countries? Are there expected similarities across health systems in the OECD? Why OECD versus the EU versus the WHO Europe Region? This is important because some of the barriers and facilitators discovered may relate to the structure and universality of primary care in the countries under scrutiny, as well as other factors.**

  

- This is a very important point and something we looked at in detail very soon after writing the original protocol.  OECD countries includes Canada, Australia, and America and when looking at the volume of papers, there was a high volume of quite diverse data.  As America and Canada have very different non-hospital-based services in place compared to most European settings, applicability to European settings was not clear. Therefore, it was decided to include European countries only and use the title ‘Member states of the European council’ which also includes the UK and Turkey. We felt it was important to include the UK and Turkey due to the concentration of refugees in both countries with Turkey holding the highest number of refugees across all of Europe. 

**5. You mentioned using** **ETHOS Typology** **as a guide for your inclusion criterion of  'homelessness', but as well as groups we traditionally consider as homeless, ETHOS includes people in accommodation for migrants, domestic violence shelters, penal institutions, couch surfing, halting sites, overcrowded settings etc., so you may need to narrow the criteria here.**

- This point will no longer be applicable as we are no longer including PEH as part of this protocol and future MMSR paper. 

# Reviewer report: Reviewer response for version 1

**DOI:** 10.21956/hrbopenres.14950.r34894

**Published:** 2023-08-31

**Authors:** Rebecca Farrington

**Affiliations:** 1 The University of Manchester, Manchester, England, UK

This is an important topic and of current relevancy. I wonder if trying to combine PEH and ASR is too big a task for one study and may result inadvertently in overgeneralisation.

 A couple of places including the abstract state that socially excluded groups present more to emergency services however on closer reading it appears to be more complex for asylum seekers. It is certainly the case in my own practice that people seeking asylum often don't present anywhere for long periods, sometimes through lack of health literacy and sometimes through fear of authorities or charging. 

 This leads me to wonder if more attention needs to be paid in the protocol to intersectionality if all three groups are being studied - homeless migrants are mentioned briefly, but I would welcome expansion on this topic. There are levels of social exclusion that are important.

 Categorisations are hard and this needs to be acknowledged. Within groups there are a range of experiences, and it can be hard to generalise - a refugee settled in a country, working and having learned a language, is in a different situation from a new migrant without status. A destitute migrant with no recourse to public funds is socially excluded in different ways from a native resident who experiences homelessness. How are you going to manage these distinctions?

Is the study design appropriate for the research question?

Partly

Is the rationale for, and objectives of, the study clearly described?

Yes

Are sufficient details of the methods provided to allow replication by others?

Yes

Are the datasets clearly presented in a useable and accessible format?

Not applicable

Reviewer Expertise:

I work with people seeking asylum and new refugees in primary care and mental health settings

I confirm that I have read this submission and believe that I have an appropriate level of expertise to confirm that it is of an acceptable scientific standard, however I have significant reservations, as outlined above.

# Response

**Published:** 2023-11-27

**Authors:** Laura Fitzharris

**Affiliations:** Trinity University, Ireland

Dear Dr. Farrington,

 Many thanks for your comments, they are greatly appreciated and we have reflected and made changes accordingly based on your feedback provided.

**1. I wonder if trying to combine PEH and ASR is too big a task for one study and may result inadvertently in overgeneralisation.**

- Yes thank you for this point and we agree, after consultation with the review team, it was agreed that it was indeed too big a scope for one review paper and may inadvertently overgeneralise these groups.  Due to the sheer high volume of research papers for these groups but more importantly that each group has unique considerations and display different patterns of health service utilisation, this would be better served in separate review papers. It was therefore decided, bearing in mind the scope of the following studies to focus on ASR only in this review. Therefore, the protocol has been amended focussing on ASR only. 

**2. On the point of socially** **excluded groups present more to emergency services however on closer reading it appears to be more complex for asylum seekers. It is certainly the case in my own practice that people seeking asylum often don't present anywhere for long periods, sometimes through lack of health literacy and sometimes through fear of authorities or charging. **

- Thank you, and we agree with this point. We have made the following points to reflect the complexity and nature of access to healthcare of this cohort in relation to accessing acute services. The following points have been added to the introduction section:

*‘Refugees and asylum seekers have been found to demonstrate different patterns of health services access and utilisation.  The health of refugees on the move in Europe is jeopardised by poor living circumstances and access to healthcare, due to language difficulties, communication, and attitudinal discrimination ^40^.*

 Later in the introduction section, the following has been added:

*‘* *A European wide systematic review by Crede et al. 2017 concluded that migrants use the ED more and somewhat differently compared to the general population.  The authors found asylum seekers and refugees tend to use emergency health services more, and for lower acuity conditions, than non-migrants.^^ It has been speculated that this is due to certain barriers accessing primary care. Research shows that ASR have diverse and additional healthcare needs to host populations. A European based literature review in this population, highlighted difficulties with communication, poor physician to patient relationships, and culturally inappropriate primary care.***** *It is also possible that ASR may avoid presentation to some health services due to fear of authorities or charging of services.’*

**3)This leads me to wonder if more attention needs to be paid in the protocol to intersectionality if all three groups are being studied - homeless migrants are mentioned briefly, but I would welcome expansion on this topic. There are levels of social exclusion that are important.**

- The protocol will no longer be based on these three groups being studied and will only include ASR. Intersectionality in relation to ASR will be considered.

**4) Categorisations are hard and this needs to be acknowledged. Within groups there are a range of experiences, and it can be hard to generalise - a refugee settled in a country, working and having learned a language, is in a different situation from a new migrant without status. A destitute migrant with no recourse to public funds is socially excluded in different ways from a native resident who experiences homelessness. How are you going to manage these distinctions?**

- Thank you for this very useful point. For the purposes of this review we included ASR only and employed strict definitions to define ASR. These definitions are used in the paper under the methodology section based on the definitions of the UNHCR. 

Of note the Inclusion criteria now narrows studies to the European setting only, ‘Members of the European Council states’, as similar factors will apply to refugees in Europe, rather than considering this more broadly in OECD countries due to the volume of papers to review. 

 At the end of the protocol the author has acknowledged the presence of subgroups within ASR; for example those who experience greater mental health difficulties and the impact of this variable on healthcare access. 
